# Effect of body surface area and gender on wall thickness thresholds in hypertrophic cardiomyopathy

**DOI:** 10.1007/s12471-019-01349-1

**Published:** 2019-11-27

**Authors:** R. Huurman, A. F. L Schinkel, N. van der Velde, D. J. Bowen, M. E. Menting, A. E. van den Bosch, M. van Slegtenhorst, A. Hirsch, M. Michels

**Affiliations:** 1 Department of Cardiology, Thorax Center, Erasmus MC, University Medical Center, Rotterdam, The Netherlands; 2 Department of Radiology and Nuclear Medicine, Erasmus MC, University Medical Center, Rotterdam, The Netherlands; 3 Department of Clinical Genetics, Erasmus MC, University Medical Center, Rotterdam, The Netherlands

**Keywords:** Hypertrophic cardiomyopathy, Family screening, Gender, Body surface area, Prediction

## Abstract

**Background:**

Family screening for hypertrophic cardiomyopathy (HCM) is based on genetic testing and clinical evaluation (maximal left ventricular wall thickness (MWT) ≥15 mm, or ≥13 mm in first-degree relatives of HCM patients). The aim of this study was to assess the effect of gender and body size on diagnosis of HCM and prediction of clinical outcome.

**Methods:**

This study includes 199 genotype-positive subjects (age 44 ± 15 years, 50% men) referred for cardiac screening. Gender-specific reference values for MWT indexed by body surface area (BSA), height and weight were derived from 147 healthy controls. Predictive accuracy of each method for HCM-related events was assessed by comparing areas under the receiver operating characteristic curves (AUC).

**Results:**

Men had a higher absolute, but similar BSA- and weight-indexed MWT compared with women (14.0 ± 3.9 mm vs 11.5 ± 3.8 mm, *p* < 0.05; 6.8 ± 2.1 mm/m^2^ vs 6.6 ± 2.4 mm/m^2^; 0.17 ± 0.06 mm/kg vs 0.17 ± 0.06 mm/kg, both *p* > 0.05). Applying BSA- and weight-indexed cut-off values decreased HCM diagnoses in the study group (48% vs 42%; 48% vs 39%, both *p* < 0.05), reclassified subjects in the largest, lightest and heaviest tertiles (≥2.03 m^2^: 58% vs 45%; ≤70 kg: 37% vs 46%; ≥85 kg: 53% vs 25%, all *p* < 0.05) and improved predictive accuracy (AUC 0.76 [95% CI 0.69–0.82] vs 0.78 [0.72–0.85]; and vs 0.80 [0.74–0.87]; both *p* < 0.05).

**Conclusions:**

In genotype-positive subjects referred for family screening, differences in MWT across gender are mitigated after indexation by BSA or weight. Indexation decreases the prevalence of HCM, particularly in larger men, and improves the predictive accuracy for HCM-related events.

## What’s New?

- Observed differences in maximal wall thickness across gender are mitigated when correcting for body size.
- Correcting for body size decreases the number of hypertrophic cardiomyopathy (HCM) diagnoses, particularly in larger men. This finding may partially explain the male predominance often seen in HCM cohorts.
- Correcting maximal wall thickness for body size and applying specific cut-off values improves the predictive accuracy for HCM-related events, suggesting superior patient identification.
- This is the first study assessing the potential role of body size indexation in the context of diagnosing HCM.

## Introduction

Hypertrophic cardiomyopathy (HCM) is the most prevalent inherited cardiac disease, with a prevalence of 0.2–0.5% in the general population [1]. Because of its familial character, and to prevent potentially life-threatening complications, family screening is advised in first-degree relatives of HCM patients. Contemporary family screening is based on genetic testing and clinical evaluation (maximal left ventricular wall thickness (MWT) ≥15 mm, or ≥13 mm in first-degree relatives of HCM patients) [2]. These absolute echocardiographic cut-off values ignore the fact that subjects differ in gender and size. Indexation is advocated in various echocardiographic measurements, particularly left ventricular and left atrial dimensions, and in the diagnosis of aortic dilatation [3, 4]. However, the role of body size indexation in HCM is unknown. The aims of this study are to assess the effect of gender and body size on the diagnosis of HCM in genotype-positive relatives, to compare this with normal values derived from healthy volunteers and to assess the effect of indexation on the prediction of HCM-related events.

## Methods

### Study population

This study included 199 first-degree genotype-positive relatives of HCM patients (99 men, mean age 44 years, range 17–82 years) referred for cardiac screening between 1995 and 2018. As all subjects had first-degree relatives with HCM, the diagnosis of HCM was based on an MWT ≥13 mm not explained by loading conditions or metabolic or mitochondrial disorders, in accordance with the guidelines [2]. The study conforms to the Declaration of Helsinki. All subjects gave informed consent and institutional review board approval was obtained.

### Genetic analysis

Genetic counselling and testing is routinely offered to HCM patients visiting our cardiogenetic outpatient clinic, and is described previously [5]. After detection of a pathogenic mutation in the proband, cascade genetic screening is offered to first-degree relatives, targeting the mutation identified in the proband. Genotype-positive relatives are then referred for cardiac screening.

### Clinical assessment

Clinical assessment included physical examination and transthoracic echocardiography (TTE). Body surface area (BSA) was calculated with the DuBois & DuBois formula [6]. TTE studies were analysed in accordance with the guidelines [2, 4]. Left ventricular wall thickness was measured in the parasternal long-axis view at or immediately below the level of the mitral valve leaflet tips, or in the parasternal short-axis view, whenever maximal wall thickness was localised to regions other than the basal segments.

### Control group

Gender-specific normal values were derived from 147 healthy volunteers (73 men, mean age 45 years). All subjects were enrolled in 2014–2015 as part of a previously published study conducted at our centre [7]. In short, healthy volunteers between 20–72 years underwent regular clinical assessment at our outpatient clinic, including TTE and height and weight measurement. Exclusion criteria were previous or existing cardiovascular disease; presence of hypertension, diabetes mellitus or hypercholesterolaemia; systemic disease or medication potentially influencing cardiac function; or cardiac abnormalities at physical examination or present on electrocardiogram. MWT was measured by an experienced sonographer (DB) and was divided by BSA for each subject. A cut-off value was derived from the mean MWT/BSA plus two standard deviations. This was repeated for height and weight separately.

### Outcome measures

As the diagnosis of HCM lacks a true gold standard, HCM-related endpoints were considered surrogate markers of disease presence, allowing comparison of the diagnostic accuracy of the different methods. These included HCM-related mortality, cardiac transplantation, implantable cardioverter-defibrillator (ICD) implantation and septal reduction therapy. Mortality was considered HCM-related in sudden cardiac death or following heart failure, stroke or an HCM intervention. Sudden cardiac death was defined as (1) instantaneous, unexpected death in patients who were previously stable, or nocturnal death with no history of worsening symptoms; (2) death following resuscitation after cardiac arrest; or (3) death following appropriate ICD intervention. Septal reduction therapy and ICD implantations were indicated in accordance with guideline recommendations [2]. Mortality data was retrieved from municipal personal records databases.

### Statistical analysis

Values were expressed as mean ± standard deviation, median [interquartile range] or number (%). Continuous variables were compared using Student’s t‑test or Mann-Whitney U test and categorical data were compared using Pearson’s χ^2^ test. Proportions of HCM diagnoses were compared using McNemar’s test. The sensitivity and specificity of the different methods were assessed using receiver operating characteristic (ROC) curves, and their predictive accuracy for HCM-related events was analysed by comparing the areas under the curve (AUC), using the* De Long* method for paired data [8]. All analyses were two-tailed and performed using SPSS version 22 (IBM Corp., Armonk, New York) and the *pROC* package for R version 3.4.1 (https://cran.r-project.org/) [9]. *P*-values <0.05 were considered statistically significant.

## Results

Baseline characteristics for controls and study patients are presented in Tab. 1. The groups were of similar age, height and BSA, but had different mean weight and MWT. In the control group, mean MWT was significantly higher in men. Men were taller, heavier and had a higher BSA. The cut-off values for MWT_BSA_, MWT_weight_ and MWT_height_ were 6.51 mm/m^2^, 0.16 mm/kg and 7.38 mm/m for men and 6.68 mm/m^2^, 0.18 mm/kg and 7.03 mm/m for women.

**Table 1:** Baseline characteristics of control and study group according to gender

Variables	Control group				Study group				
	Overall	Male	Female	Male vs female, controls	Overall	Male	Female	Male vs female, study group	Control vs study group
	(*n* = 147)	(*n* = 73)	(*n* = 74)		(*n* = 199)	(*n* = 99)	(*n* = 100)		
Age, *y*	45 ± 14	44 ± 14	45 ± 14	0.582	44 ± 15	43 ± 14	46 ± 15	0.105	0.728
Weight, *kg*	75 ± 13	82 ± 11	69 ± 9	<0.001	78 ± 16	87 ± 14	70 ± 13	<0.001	0.043
Height, cm	175 ± 9	181 ± 10	169 ± 10	<0.001	174 ± 10	182 ± 7	167 ± 6	<0.001	0.623
BSA, *m*^*2*^	1.89 ± 0.19	2.03 ± 0.15	1.76 ± 0.1	<0.001	1.92 ± 0.23	2.07 ± 0.17	1.78 ± 0.18	<0.001	0.189
MWT, *mm*	9.2 ± 1.7	9.9 ± 1.6	8.6 ± 1.6	<0.001	12.8 ± 4.0	14.0 ± 3.9	11.5 ± 3.8	<0.001	<0.001
MWT/BSA, *mm/m*^*2*^	4.9 ± 0.9	4.9 ± 0.8	4.9 ± 0.9	0.939	6.7 ± 2.3	6.8 ± 2.1	6.6 ± 2.4	0.358	<0.001
MWT/weight, *mm/kg*	0.13 ± 0.02	0.12 ± 0.02	0.13 ± 0.03	0.030	0.17 ± 0.06	0.17 ± 0.06	0.17 ± 0.06	0.907	<0.001
MWT/height, *mm/m*	5.3 ± 0.98	5.5 ± 0.96	5.01 ± 0.97	0.019	7.3 ± 2.3	7.8 ± 2.2	6.9 ± 2.3	0.012	<0.001

Data are expressed as mean ± standard deviation

*BSA* body surface area; *MWT* maximal wall thickness

### Reclassification of subjects

**Fig. 1:**
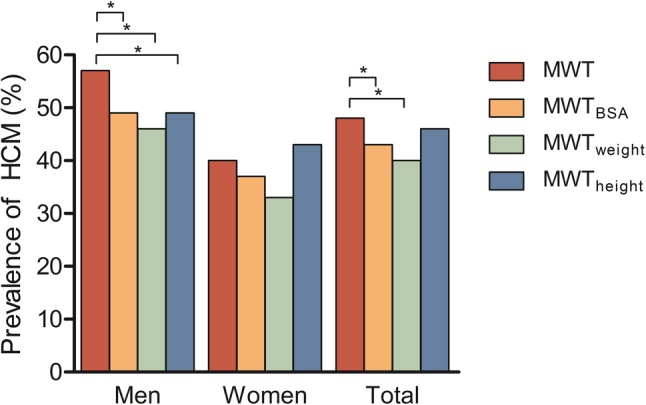
Prevalence of HCM before and after indexation stratified by gender. Indexation by BSA or weight significantly decreased diagnoses in men and in the total group. Asterisk denotes significant differences between proportions. *HCM* hypertrophic cardiomyopathy, *MWT* maximal wall thickness, *BSA* body surface area

In the study group, 56 (57%) men and 40 (40%) women were diagnosed with HCM according to the absolute cut-off. The impact of indexed cut-offs on the proportion of HCM diagnoses is illustrated in Fig. 1. After BSA indexation, the prevalence of HCM decreased significantly, with 14 subjects (8 men) reclassified as no HCM, and 4 subjects (1 man) reclassified as HCM. The decrease in diagnoses remained significant for men separately, but not for women. A similar pattern was seen after indexation by weight, but not after indexation by height. Subjects reclassified as no HCM after indexation by BSA had a mean BMI of 28.8 ± 3.3 kg/m^2^, compared with 21.3 ± 2.3 kg/m^2^ for subjects reclassified as HCM and 25.6 ± 4.1 kg/m^2^ for non-reclassified subjects.

### Effect of body size on diagnosis of HCM

**Fig. 2:**
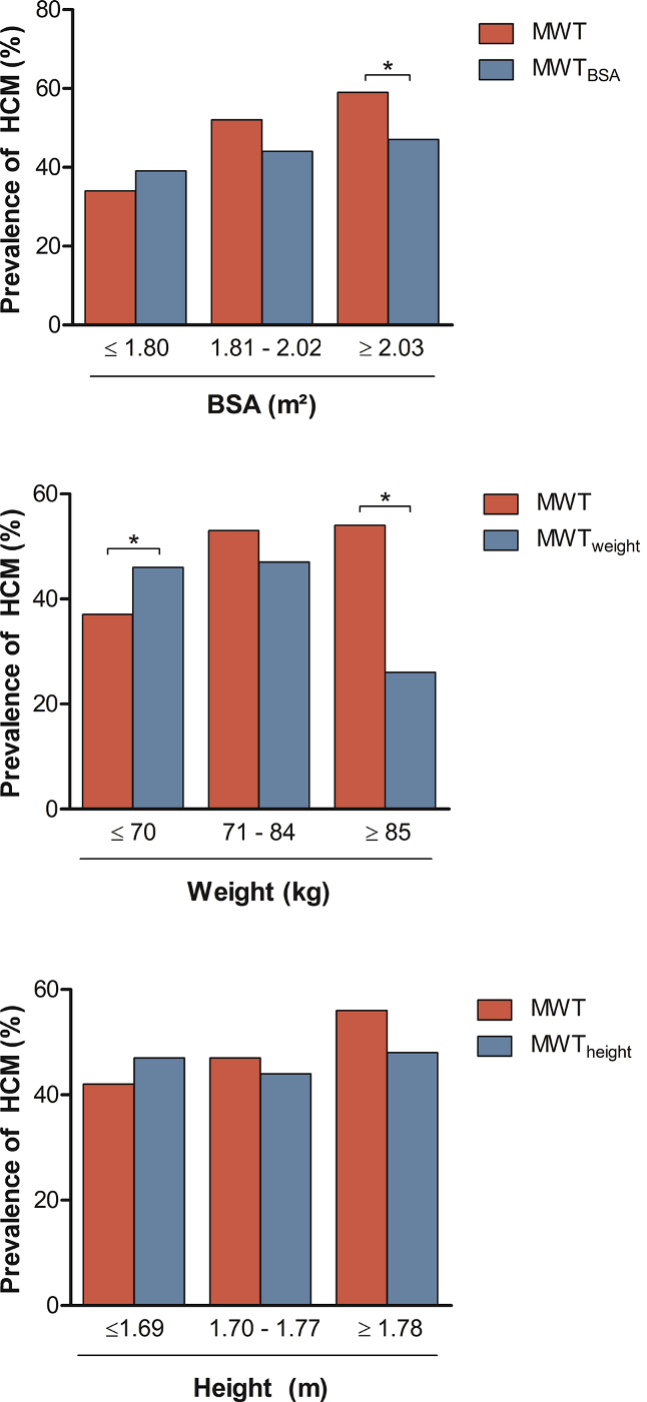
Prevalence of HCM diagnoses in study group, after indexation according to **a** BSA, **b** weight and **c** height. Significant differences in proportions of HCM were seen in the highest BSA tertile, after indexation for BSA, and in the lowest and highest weight tertiles after indexation for weight. Asterisk denotes significant differences between proportions. *BSA* body surface area, *HCM* hypertrophic cardiomyopathy, *MWT* maximal wall thickness

Subjects were stratified by BSA tertiles and the prevalence of HCM using the conventional and BSA-indexed method was compared in each tertile (Fig. 2a). Larger patients were diagnosed less often (59% vs 47%, *p* = 0.008). Subjects in the lowest weight tertile were diagnosed less often after indexation by weight. The opposite was true for the highest tertile (normal vs indexed, ≤70 kg: 37% vs 46%, *p* = 0.03; ≥85 kg: 54% vs 26%, *p* < 0.001, Fig. 2b). No significant changes were seen after indexation by height (Fig. 2c).

### Clinical outcome

**Fig. 3:**
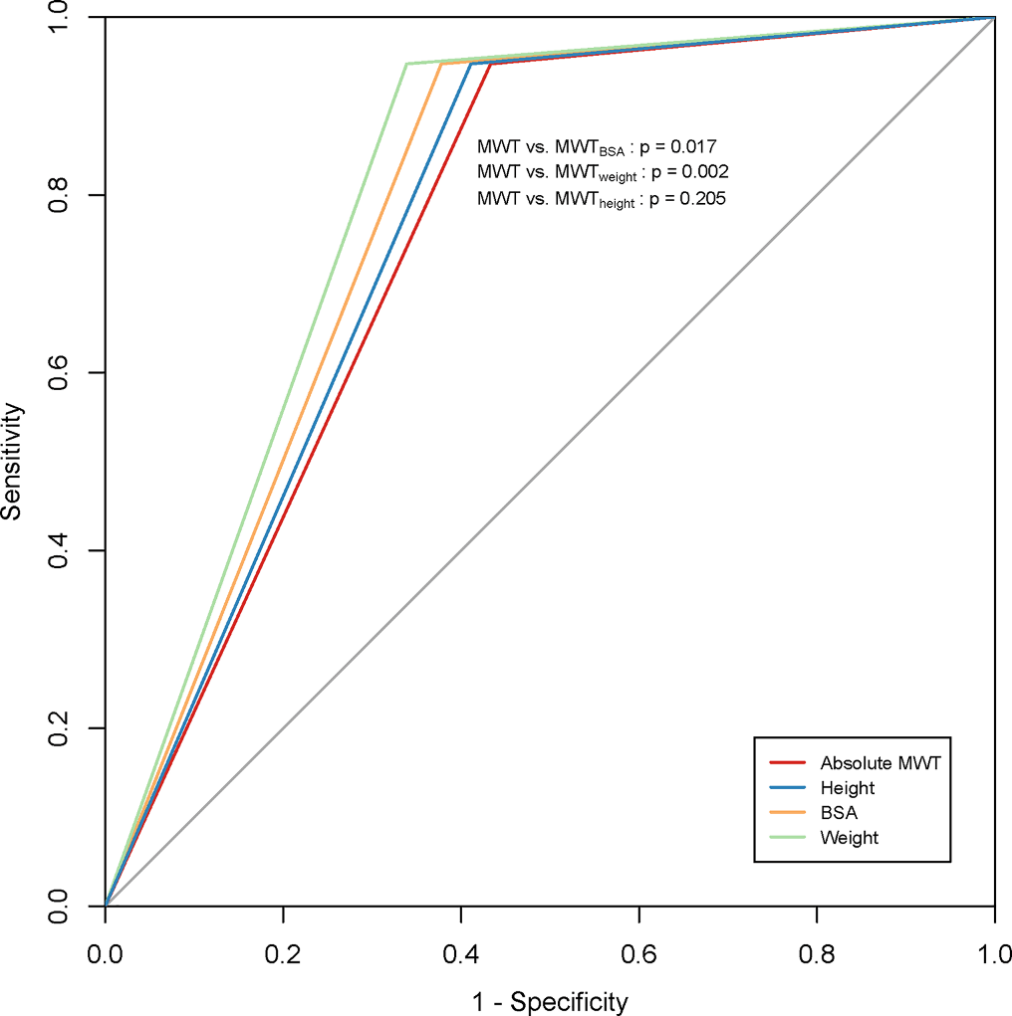
Receiver operating characteristic curves illustrating predictive accuracy of all diagnostic methods for HCM-related events. A significant increase in the AUC was seen using BSA- or weight-indexed values: difference between areas was 0.028 [95% CI 0.005–0.051] for MWT and MWT_BSA_, 0.047 [0.017–0.078] for MWT and MWT_weight_ and 0.011 [−0.006–0.028] for MWT and MWT_height_. *HCM* hypertrophic cardiomyopathy, *AUC* area under the curve, *BSA* body surface area, *MWT* maximal wall thickness

In the study group, 23 events occurred in 19 patients, over a median follow-up of 5.8 [2.3–8.8] years. Sudden cardiac death occurred in 2 patients and there was 1 cardiac transplantation. Five patients underwent myectomy and ICDs were implanted in 15 patients. There were no events in patients reclassified from HCM to no HCM and vice versa. Predictive accuracy for HCM-related events improved significantly after indexation by BSA and weight (AUC for MWT: 0.757 [95% CI 0.69–0.82]; MWT_BSA_: 0.785 [95% CI 0.72–0.85], *p* < 0.05; MWT_weight_: 0.804 [95% CI 0.74–0.87], *p* < 0.01, Fig. 3), but was similar after indexation by height (MWT_height_: 0.768 [95% CI 0.71–0.83], *p* = 0.2). Indexation by BSA, weight and height increased specificity from 0.57 to 0.62, 0.66 and 0.59 respectively, whilst sensitivity was 0.947 for all methods.

## Discussion

The main findings of this study are that in genotype-positive subjects presented for cardiac screening: 1) mean MWT is higher in men than in women, and that this difference is nullified after indexation by BSA or weight; 2) body size influences the diagnosis of HCM, with the largest and heaviest subjects being diagnosed significantly less often after BSA- or weight-indexation and application of gender-specific reference values obtained in healthy controls, with the reverse being true for the lightest tertile; 3) weight is the primary determinant of these differences; and 4) indexation by BSA or weight augments the predictive accuracy for HCM-related events, suggesting superior patient identification using gender-specific cut-off values for MWT_BSA_ (men: 6.51 mm/m^2^, women: 6.68 mm/m^2^) and MWT_weight_ (men: 0.16 mm/kg, women: 0.18 mm/kg), instead of the conventional cut-off.

Indexation is increasingly being used in the context of cardiac disease [3, 4]. Left ventricular mass, calculated using septal and posterior wall dimensions, is routinely reported after correcting for BSA. Nevertheless, there is no mention of body size when reporting wall thickness alone. In this cohort, the diagnosis of HCM was predominantly altered in patients with a high BSA, but also in the lightest and heaviest group when stratifying by weight alone. The decrease of diagnoses in men, being taller and heavier than women, supports the notion that body size matters in diagnosing HCM.

Furthermore, our results imply that these differences are driven by weight variations, which is illustrated by the effects of indexation by BSA and weight and the lack thereof when indexing by height. Indexation by weight reclassified a significant number of the lightest and heaviest subjects, and subjects reclassified as no HCM were predominantly obese. Left ventricular enlargement is a well-recognised physiological adaptation aimed at optimising stroke volume to compensate for an increased oxygen demand [10–13], and is also seen in HCM [14]. Olivotto *et al. *studied 275 HCM patients (age 48 ± 14 years; 70% male) and found that BMI was independently associated with magnitude of hypertrophy, defined as an increase in likelihood of having a left ventricular mass in the highest quartile (>120 g/m^2^) [14].

The autosomal dominant inheritance pattern of HCM implies an equal gender distribution. However, many cohorts illustrate a male predominance [15–20], bringing forth potential explanations ranging from delays in clinical presentation to differences in gene expression and sex hormone receptor levels [18]. In this cohort, less men, but not women, were diagnosed with HCM after indexation, suggesting that applying non-indexed cut-off values overestimates the prevalence of HCM in men. Moreover, in the control group, MWT was higher in men than in women. Although it is unlikely that this finding is the primary cause of the observed gender disparity, especially in light of the aforementioned hypotheses, it does offer a partial explanation.

Additionally, gender disparities are reported when comparing disease severity and outcome [15, 21–25], generally in favour of men. In a cohort of 27 women and 44 men, Nijenkamp *et al. *demonstrated that tissue samples of women who underwent myectomy showed decreased expression of phospholamban (PLN) and sarco/endoplasmic reticulum Ca^2+^ ATPase (SERCA2), more compliant titin and more fibrosis [22]. Absolute MWT, left ventricular and left atrial diameter were similar across gender, but BSA-corrected values were higher in women. Although we are unable to corroborate these results, our findings do support indexation when assessing gender differences.

The clinical relevance of indexation in HCM is demonstrated by the augmented predictive accuracy for HCM-related events after indexation for BSA or weight, by improving specificity without sacrificing sensitivity, indicating proper identification of HCM patients. It is reassuring that no endpoints occurred in HCM patients reclassified as no HCM after a median follow-up of 5.8 years. To our knowledge, this is the first study demonstrating the value of indexation in HCM, albeit in a single centre and with a modest size and low event rate. As the clinical spectrum of HCM is expanding with the advent of advanced genetic testing and imaging, the prevalence of HCM will increase, emphasising the importance of correct patient identification as a means of decreasing the burden on health care systems [1, 26, 27], by virtue of less frequent follow-up and a decreased need for cardiac monitoring, exercise testing and imaging. Additionally, a reduction in HCM diagnoses has the potential to preclude patients from unnecessary (exercise) restrictions, simultaneously decreasing their psychological burden. Future studies in index patients (i.e. those requiring MWT measurement ≥15 mm) will potentially decrease HCM diagnoses in that group, leading to less (unnecessary) genetic testing and family screening.

This study has several limitations. In the first place, the inherent limitations of retrospective studies apply here, although some of these limitations were attenuated by a dedicated assessment of MWT in controls. Moreover, scaling cardiac size using BSA in patients with hypertensive left ventricular hypertrophy has been shown to underestimate the prevalence of left ventricular hypertrophy in obese individuals [28], raising the possibility that the same problem occurs in our data. As there is a paucity of data on the relationship between body size and cardiac dimensions in the context of HCM, we cannot exclude the influence of obesity on our results and acknowledge that the concept of scaling MWT to body size requires further attention before it can be incorporated into clinical practice. Furthermore, body size is only one factor potentially influencing HCM diagnoses, with an individual’s prior chance of HCM likely depending on several other components (e.g. hypertension, engaging in strenuous physical activity). This includes inter-reader and intra-reader variability, which can potentially influence HCM diagnoses in our data as well as in clinical practice. Finally, although mortality data was complete for all patients, we cannot exclude the possibility of other events (mainly ICD implantations) occurring in other centres.

In conclusion, in genotype-positive subjects referred for cardiac screening, differences in MWT across gender are mitigated after correcting for body size, especially body weight. The effect of body size on the diagnosis of HCM is demonstrated by the reclassification of mainly male subjects when indexing by BSA or body weight. The improved predictive accuracy for HCM-related events after indexation by BSA or weight suggests that correcting for body size has a potential role in the diagnosis of HCM.
